# Vacuoles, E1 Enzyme, X-linked, Autoinflammatory, Somatic (VEXAS) Syndrome With Multisystem Involvement: Imaging and Genetic Insights From a Case Report

**DOI:** 10.7759/cureus.85182

**Published:** 2025-06-01

**Authors:** Lauren E Arsenault, Sumeet Virmani, Pokhraj P Suthar

**Affiliations:** 1 Department of Diagnostic Radiology and Nuclear Medicine, Rush University Medical Center, Chicago, USA

**Keywords:** ct, inflammation, management, mri, vexas syndrome

## Abstract

Vacuoles, E1 enzyme, X-linked, autoinflammatory, somatic (VEXAS) syndrome is a recently recognized, life-threatening autoinflammatory disorder caused by somatic mutations in the *UBA1* gene, resulting in dysregulated innate immune responses. It predominantly affects older males and is characterized by systemic inflammation involving multiple organ systems. We report the case of an 85-year-old man with recurrent inflammation affecting the ears, nose, skin, lungs, and hematologic system. Laboratory tests revealed cytopenias and elevated inflammatory markers, while imaging showed cartilaginous inflammation and pulmonary infiltrates. Bone marrow biopsy demonstrated vacuolated myeloid precursors, and genetic testing confirmed a *UBA1* mutation (p.Met41Val), establishing the diagnosis. Treatment with tocilizumab and corticosteroids led to marked clinical improvement. This case highlights the importance of considering VEXAS syndrome in patients with unexplained systemic inflammation and hematologic abnormalities to ensure timely diagnosis and appropriate management.

## Introduction

Vacuoles, E1 enzyme, X-linked, autoinflammatory, somatic (VEXAS) syndrome is a recently recognized hemato-inflammatory disorder first described in 2020 [1]. It poses a significant diagnostic challenge due to its broad and overlapping clinical features [1]. Affected patients often present with systemic inflammation, including recurrent fevers, cytopenias, painful rashes, thromboembolic events, respiratory symptoms, and chondritis, closely mimicking various rheumatologic, hematologic, and infectious diseases [2,3].

The syndrome is driven by somatic mutations in the *UBA1* gene within hematopoietic progenitor cells, leading to dysregulated innate immunity, vacuolization of bone marrow precursors, and progressive inflammatory damage [1,4]. Diagnosis typically requires comprehensive clinical evaluation, laboratory tests, imaging studies, and ultimately, genetic confirmation. VEXAS is also considered a premalignant condition, with potential progression to myelodysplastic, myeloproliferative, or lymphoproliferative disorders [3,5].

Despite likely being underrecognized, emerging data suggest that VEXAS may be more prevalent than many rare autoimmune diseases [6]. This case underscores the importance of including VEXAS syndrome in the differential diagnosis of patients with unexplained systemic inflammation and cytopenias. It also aims to raise awareness among clinicians and radiologists regarding the imaging findings and diagnostic considerations associated with this evolving entity.

## Case presentation

An 85-year-old male presented with a longstanding history of relapsing, unexplained inflammatory symptoms involving multiple organ systems. In the months prior to presentation, he experienced recurrent facial and cervical swelling, auricular and nasal chondritis, progressive respiratory compromise, cutaneous manifestations, and bilateral sensorineural hearing loss. These symptoms were initially managed under the presumptive diagnosis of a systemic vasculitis or connective tissue disorder.

On physical examination, the patient was alert, afebrile, and hemodynamically stable, appearing in no acute distress. Examination of the head and neck revealed mild residual swelling over the left cheek and thickening of the nasal cartilage. The auricular cartilage was firm and mildly tender bilaterally. Pulmonary auscultation revealed faint bibasilar crackles without wheezing. Cardiovascular examination was unremarkable, aside from distant heart sounds. The abdominal examination was benign, and there was no peripheral edema or lymphadenopathy. Neurological examination was nonfocal, and no inguinal lymphadenopathy was noted.

Laboratory findings during this episode included a markedly elevated C-reactive protein (212.6 mg/L), persistent normocytic anemia (hemoglobin 9.8 g/dL), leukopenia (2.74 × 10³/μL), and neutropenia (1.61 × 10³/μL), with a normal platelet count (188 × 10³/μL). Transient hyperglycemia (fasting glucose 239 mg/dL) was observed, likely attributable to corticosteroid therapy and dietary indiscretion (Table 1). The persistence of elevated inflammatory markers despite antimicrobial therapy, along with hematologic abnormalities - including normocytic anemia, leukopenia, and neutropenia - prompted further immunologic and genetic evaluation.

**Table 1:** Laboratory values

Test	Patient value	Normal reference range
C-reactive protein	212.6 mg/L	<5 mg/L
Hemoglobin	9.8 g/dL	Male: 13.5-17.5 g/dL
White blood cell count	2.74 × 10³/μL	4.0-11.0 × 10³/μL
Absolute neutrophil count	1.61 × 10³/μL	1.8-7.5 × 10³/μL
Platelet count	188 × 10³/μL	150-450 × 10³/μL
Fasting glucose	239 mg/dL	70-99 mg/dL

A peripheral blood smear revealed vacuolated myeloid precursors, and targeted genetic testing identified a somatic pathogenic variant in the *UBA1* gene (p.Met41Val), confirming the diagnosis of VEXAS syndrome. The diagnosis was made in accordance with current consensus criteria, integrating clinical features with molecular confirmation of the *UBA1 *mutation.

**Figure 1:**
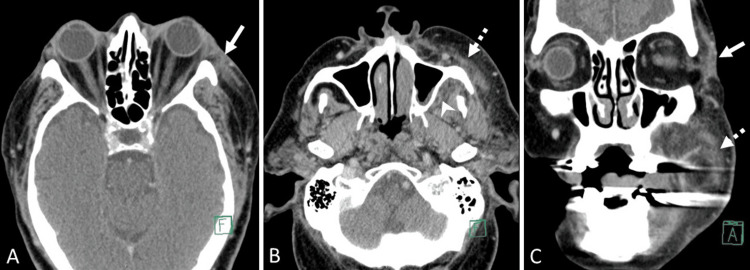
Contrast-enhanced CT of the paranasal sinuses (A, B) Axial and (C) coronal images show mild soft tissue thickening and fat stranding in the left buccal space (dashed white arrow), retroantral regions (solid white arrowhead), and left inferior periorbital soft tissues (solid white arrow). These findings are suggestive of cellulitis.

**Figure 2:**
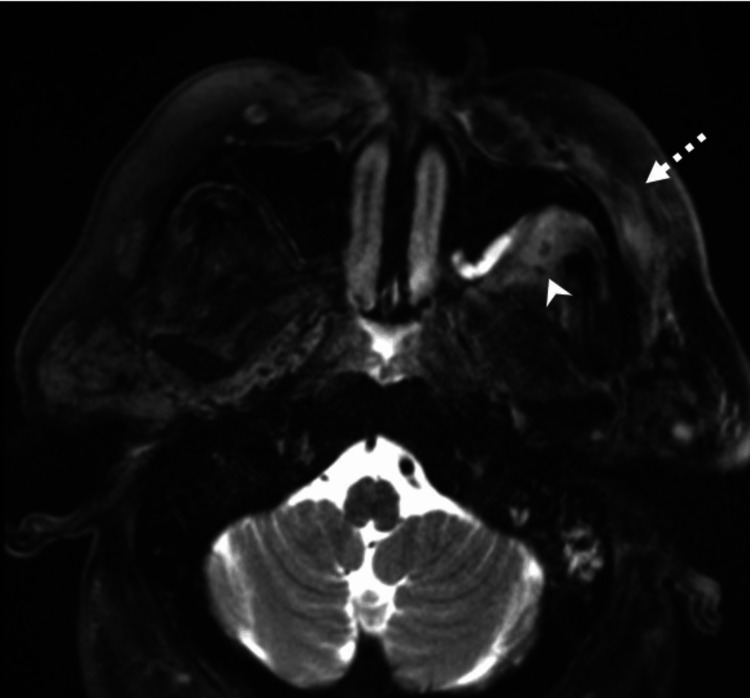
MRI of the paranasal sinuses Axial T2-weighted MRI image shows diffuse soft tissue edema involving the left buccal space (dashed white arrow), left pterygopalatine fossa, and retroantral region (white arrowhead). These findings are most consistent with cellulitis.

Contrast-enhanced CT images revealed mild soft tissue thickening and fat stranding involving the left buccal space, retroantral region, and left inferior periorbital soft tissues, suggestive of early cellulitis without abscess formation (Figure 1). MRI of the paranasal sinuses further demonstrated diffuse soft tissue edema in the left buccal space, pterygopalatine fossa, and retroantral region, consistent with cellulitis. No sizable fluid collections were identified (Figure 2).

**Figure 3:**
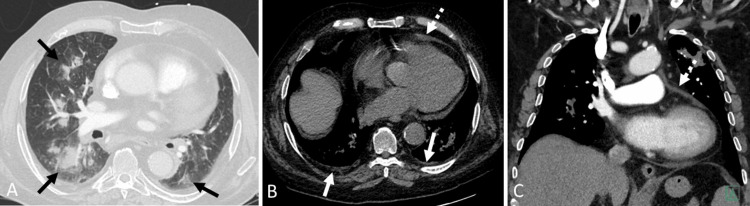
Chest CT (A) Axial lung window image reveals patchy consolidative opacities with areas of ground-glass attenuation in both lungs, consistent with neutrophilic alveolitis (black arrows). (B) Axial and (C) coronal soft tissue window images show mild pericardial effusion (dashed white arrow) and trace bilateral pleural effusions (solid white arrow).

Chest CT showed multifocal, patchy consolidative opacities accompanied by areas of ground-glass attenuation in both lungs. These findings were suggestive of an inflammatory process, with a pattern consistent with neutrophilic alveolitis. The distribution and appearance raised concern for an underlying autoinflammatory or immune-mediated etiology. Additionally, mild pericardial effusion and trace bilateral pleural effusions were noted, further supporting systemic inflammation with multiorgan involvement (Figure 3). 

**Figure 4:**
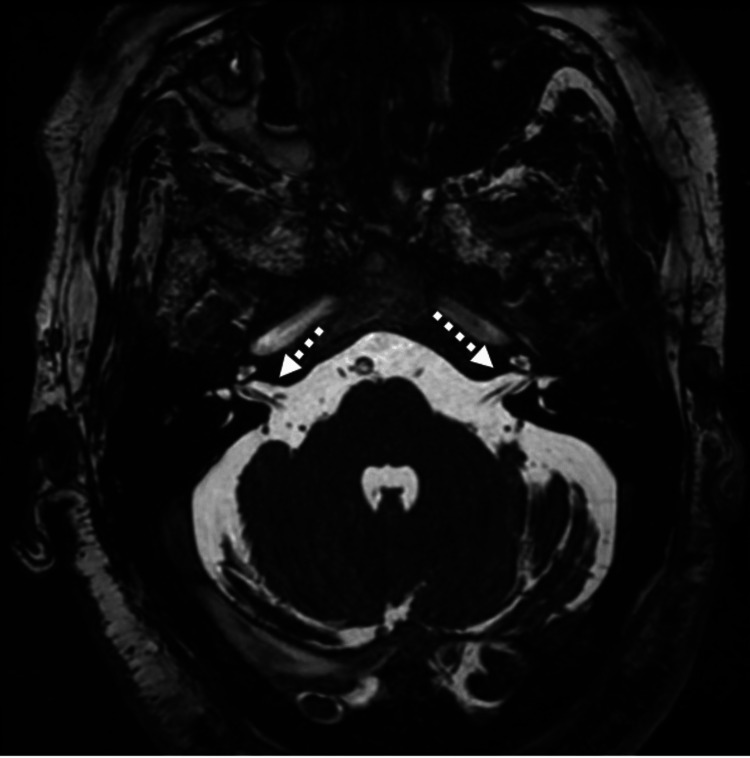
MRI of the temporal bones Axial 3D FIESTA MRI demonstrates normal bilateral internal auditory canals and inner ear structures (dashed white arrows), with no abnormal enhancement or pathological findings.

MRI of the temporal bones demonstrated unremarkable bilateral internal auditory canals and inner ear structures, with no evidence of abnormal enhancement or infection (Figure 4).

The identification of the *UBA1* mutation through next-generation sequencing, along with characteristic hematologic findings such as cytopenias and vacuolization, and evidence of multi-organ inflammatory involvement, confirmed the diagnosis of VEXAS syndrome. Recognizing the protean features of VEXAS is essential for timely diagnosis and appropriate management, particularly as awareness of its diverse organ involvement, including genitourinary inflammation, continues to expand. The patient was treated with intravenous tocilizumab infusions and prednisolone. At the three-month follow-up, he remained clinically stable, with symptoms well controlled on the current treatment regimen.

## Discussion

Overview and diagnostic challenges

VEXAS syndrome, first described in 2020, poses a significant diagnostic challenge due to its heterogeneous clinical manifestations and the absence of disease-specific imaging markers [1]. It primarily affects older males and is characterized by systemic inflammation involving multiple organ systems. Patients typically present with a constellation of symptoms including recurrent fevers, cytopenias, painful cutaneous rashes, thromboembolic events, respiratory symptoms, and chondritis - features that closely mimic various autoimmune, hematologic, and infectious diseases [3]. This clinical overlap, coupled with a lack of specific biomarkers, often results in delayed or missed diagnoses. As such, a thorough diagnostic approach incorporating multiple imaging modalities and, ultimately, genetic testing is essential.

Clinical manifestations

The clinical presentation of VEXAS syndrome reflects its dual inflammatory and hematologic nature. Systemic symptoms commonly include persistent fever, fatigue, and malaise. Hematologic abnormalities such as normocytic anemia, neutropenia, and, less commonly, thrombocytopenia contribute to fatigue, increased susceptibility to infections, and bleeding tendencies [4]. Chondritis, particularly involving the auricles and nasal cartilage, is a hallmark feature, often presenting with painful swelling and erythema that may progress to cartilage deformities. Relapsing polychondritis is frequently observed within the disease spectrum.

Cutaneous manifestations such as rashes and purpura are believed to result from systemic inflammation and thrombocytopenia. Respiratory involvement may be due to neutrophilic alveolitis or interstitial lung disease, with imaging often revealing patchy consolidations, ground-glass opacities, and pleural effusions. Thromboembolic events, including deep vein thrombosis and pulmonary embolism, add further complexity to the clinical course. Neurologically, bilateral sensorineural hearing loss is relatively common and often associated with auricular chondritis; focal neurological deficits have also been reported.

**Figure 5:**
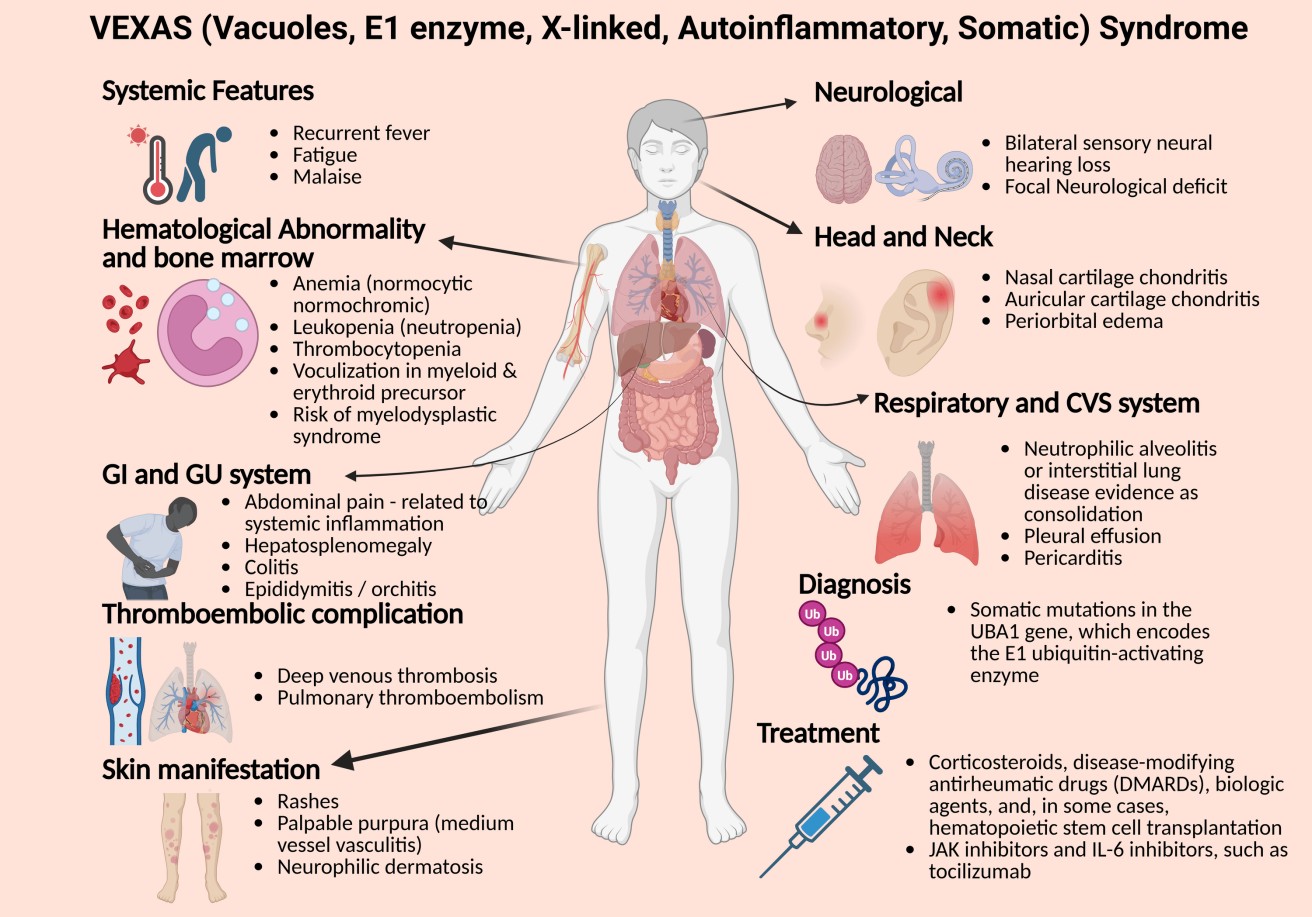
Illustration of the multisystem clinical features of VEXAS syndrome This graphic highlights the diverse organ involvement in VEXAS syndrome, along with key elements of diagnosis and treatment. Created in BioRender by Pokhraj P. Suthar (https://BioRender.com/top0q8o)

Bone marrow involvement provides a key diagnostic clue, with vacuolization of myeloid and erythroid precursors frequently observed, often preceding the onset of myelodysplastic or myeloproliferative disorders [4]. Gastrointestinal symptoms such as abdominal pain can also occur, typically in the setting of systemic inflammation (Figure 5).

Pathophysiology

Due to its highly variable clinical presentation, early recognition and genetic testing are crucial for accurate diagnosis and management [3,5-7]. VEXAS syndrome is caused by somatic mutations in the *UBA1* gene, which encodes the E1 enzyme essential for the ubiquitination pathway. These mutations impair ubiquitin-mediated protein signaling, leading to dysregulated innate immune responses and systemic inflammation. The mutation arises in hematopoietic progenitor cells, resulting in clonal expansion of mutant myeloid cells that drive both inflammation and hematologic abnormalities. The X-linked nature of the mutation explains the strong male predominance [5,8].

Differential diagnosis

The multisystem involvement of VEXAS syndrome often leads to misdiagnosis, as its features overlap with several inflammatory diseases, including systemic lupus erythematosus (SLE), relapsing polychondritis, and cutaneous polyarteritis nodosa [9]. Distinguishing VEXAS from these conditions is essential:

SLE commonly presents with joint pain, cutaneous involvement, fatigue, and organ manifestations, including the lungs and kidneys. While inflammatory markers are elevated and anemia is often present, SLE lacks the hallmark bone marrow vacuolization seen in VEXAS. Additionally, the presence of neutrophilic alveolitis, periorbital soft tissue swelling, and typically spared renal involvement helps differentiate VEXAS from SLE [10].

Relapsing polychondritis shares overlapping symptoms such as auricular and nasal chondritis and joint inflammation. However, VEXAS is more likely to present with recurrent thromboembolic events and neutrophilic alveolitis - findings generally absent in relapsing polychondritis. Hematologic abnormalities and bone marrow vacuolization are also uncommon in the latter [11].

Cutaneous polyarteritis nodosa typically manifests with nodular skin lesions, fatigue, and joint pain. Respiratory involvement is rare, and features such as pleural or pericardial effusions and auricular swelling are not commonly seen. Absence of bone marrow changes and a *UBA1 *mutation further distinguishes it from VEXAS [12].

Management and treatment

The primary treatment goal in VEXAS syndrome is to suppress systemic inflammation and manage hematologic dysfunction. Corticosteroids are often the first line of therapy, though patient responses can vary [6,13]. Traditional immunosuppressants, such as conventional disease-modifying antirheumatic drugs, have shown limited efficacy. However, emerging evidence supports the use of biologic therapies, particularly JAK inhibitors and IL-6 inhibitors like tocilizumab [14]. In our case, the patient received intravenous tocilizumab along with prednisolone, which led to sustained clinical improvement during follow-up.

For severe or treatment-refractory cases, hematopoietic stem cell transplantation is being explored as a potential curative option, though further data are needed to establish its role.

## Conclusions

VEXAS syndrome is a newly recognized, potentially life-threatening hemato-inflammatory disorder that requires heightened clinical awareness due to its overlapping features with other autoimmune and hematologic diseases. This case emphasizes the need to consider VEXAS in older men presenting with unexplained systemic inflammation, cytopenias, and relapsing chondritis. Key diagnostic clues include bone marrow vacuolization, imaging findings of systemic inflammation, and confirmation of a somatic *UBA1 *mutation.

Early and accurate diagnosis requires a multidisciplinary approach involving rheumatologists, hematologists, pulmonologists, radiologists, infectious disease specialists, and geneticists. Immunomodulatory therapies, such as intravenous tocilizumab at 8 mg/kg every four weeks combined with corticosteroids, have shown promising results in improving clinical outcomes. Given the syndrome’s premalignant nature and potential for rapid progression, timely identification and intervention are critical. Ongoing research is needed to refine diagnostic criteria and develop targeted therapies that can improve long-term prognosis.
